# RNA structure determination: From 2D to 3D

**DOI:** 10.1016/j.fmre.2023.06.001

**Published:** 2023-06-12

**Authors:** Jie Deng, Xianyang Fang, Lin Huang, Shanshan Li, Lilei Xu, Keqiong Ye, Jinsong Zhang, Kaiming Zhang, Qiangfeng Cliff Zhang

**Affiliations:** a Guangdong Provincial Key Laboratory of Malignant Tumor Epigenetics and Gene Regulation, Guangdong-Hong Kong Joint Laboratory for RNA Medicine, Sun Yat-Sen Memorial Hospital, Sun Yat-Sen University, Guangzhou 510120, China; b Beijing Frontier Research Center for Biological Structure, Center for Synthetic and Systems Biology, School of Life Sciences, Tsinghua University, Beijing 100084, China; c Key Laboratory of RNA Biology, CAS Center for Excellence in Biomacromolecules, Institute of Biophysics, Chinese Academy of Sciences, Beijing 100101, China; d MOE Key Laboratory for Cellular Dynamics and Center for Advanced Interdisciplinary Science and Biomedicine of IHM, Division of Life Sciences and Medicine, University of Science and Technology of China, Hefei 230027, China; e University of Chinese Academy of Sciences, Beijing 100049, China; f MOE Key Laboratory of Bioinformatics, Beijing Advanced Innovation Center for Structural Biology & Frontier Research Center for Biological Structure, Center for Synthetic and Systems Biology, School of Life Sciences, Tsinghua University, Beijing 100084, China; g Tsinghua-Peking Center for Life Sciences, Beijing 100084, China

**Keywords:** RNA structure, RNA structure probing, X-ray crystallography, Nuclear magnetic resonance spectroscopy, Cryo-electron microscopy, Small angle X-ray scattering

## Abstract

RNA molecules serve a wide range of functions that are closely linked to their structures. The basic structural units of RNA consist of single- and double-stranded regions. In order to carry out advanced functions such as catalysis and ligand binding, certain types of RNAs can adopt higher-order structures. The analysis of RNA structures has progressed alongside advancements in structural biology techniques, but it comes with its own set of challenges and corresponding solutions. In this review, we will discuss recent advances in RNA structure analysis techniques, including structural probing methods, X-ray crystallography, nuclear magnetic resonance, cryo-electron microscopy, and small-angle X-ray scattering. Often, a combination of multiple techniques is employed for the integrated analysis of RNA structures. We also survey important RNA structures that have been recently determined using various techniques.

## 1 Introduction

For a considerable period, RNA was primarily regarded as a passive messenger that facilitated the transfer of genetic information from DNA into protein. However, the discovery of numerous non-coding RNAs (ncRNAs) in recent decades has revolutionized our understanding of RNA's functional diversity. It is now evident that RNA not only acts as a template for protein, RNA, or DNA synthesis but also serves as a catalytic center for RNA splicing and protein translation. Moreover, RNA plays a regulatory role in a wide array of biological processes, including epigenetic modification, DNA replication, transcription, splicing, translation, as well as glucose and lipid metabolism [[1], [2]]. Perturbations in RNA processing and metabolism can lead to human diseases [[3], [4]]. Therefore, it is crucial to comprehensively investigate the molecular mechanisms that underlie RNA functions to develop innovative therapeutics.

The functional versatility of RNA is closely linked to its ability to form intricate and hierarchical structures. Similar to protein structure, RNA structure can be described in hierarchical terms: primary, secondary, tertiary, and quaternary structures. While smaller ncRNAs consisting of less than 40 nucleotides (nt), such as microRNA (miRNA), small interfering RNA (siRNA), and piwi-interacting RNA (piRNA), mainly rely on their primary sequences to target complementary molecules and form RNA-protein complexes, larger RNAs tend to adopt complex secondary and tertiary structures to carry out their functions. A notable example is tRNA, which exhibits a well-known cloverleaf 2-dimensional (2D) structure and a distinctive l-shaped 3-dimensional (3D) structure. Additionally, other RNAs, such as catalytic ribozymes, riboswitches and regulatory elements found in mRNAs and viral RNAs, have well-defined 3D structures. Understanding the structure and interactions of RNAs is essential for comprehending their functions and developing RNA-targeted or RNA-based pharmaceuticals [5].

RNA is composed of nucleotides that can adopt either single-stranded or double-stranded structures, resulting in a diverse array of secondary structure elements, including stems, hairpins, bulges, internal loops, junctions, and pseudoknots. These secondary structure elements can interact with each other, leading to the formation of more intricate tertiary structures. The secondary structure of RNA can be predicted computationally using score-based or machine-learning methods [6]. Experimental techniques such as chemical or enzymatic probing, in conjunction with deep-sequencing techniques, enable the measurement of RNA's secondary structure at the transcriptome level. To determine the 3D structures of RNA, various biophysical approaches are employed, including X-ray crystallography (XRC), nuclear magnetic resonance (NMR) spectroscopy, cryo-electron microscopy (cryo-EM), and small angle X-ray scattering (SAXS). Typically, the initial step in RNA structure analysis involves obtaining 2D structure information, which serves as a foundation for studying its corresponding 3D structure.

The first RNA 3D structure, which was the yeast tRNA^Phe^, was determined by X-ray crystallography in the 1970s [[7], [8]]. As of October 26, 2022, there are currently 1663 RNA-only and 4588 RNA-protein complexes structures deposited in the Protein Structure Database (PDB). These structures have greatly enhanced our understanding of RNA folding and functional capabilities. However, it is important to note that our knowledge about RNA 3D structure is still relatively limited compared to proteins, which make up approximately 90% of the PDB entries. This disparity is not due to a lack of importance or interest in RNA, but rather because studying the 3D structures of RNA poses greater challenges compared to proteins, primarily due to the inherent flexibility of RNA molecules. Nevertheless, over the past few decades, there has been steady progress in studying RNA 3D structure, driven by advancements in structural biology techniques and the discovery of new RNA molecules. In this review, we will discuss the latest advancements in techniques used to analyze 2D and 3D structures of RNA, including various RNA structure probing methods, XRC, NMR, cryo-EM, and SAXS. While a recent comprehensive review has already discussed techniques for probing the RNA “structurome” [9], we will provide a more in-depth discussion of biophysical methods used to study RNA 3D structures and focus on recent improvements in probing methods. Furthermore, we will provide a summary of important RNA tertiary structures that have been recently determined using these respective techniques (Table S1).

## 2 RNA structure probing methods

Probing methods for studying the secondary and tertiary structure of RNA were first developed around four decades ago [10]. These methods rely on enzymatic or chemical probes that react differentially with single- or double-stranded nucleotides of an RNA molecule, which leads to the cleavage of RNAs or modification of nucleotides. Originally, these cleavages or modifications were detected using primer extension and gel electrophoresis, which limited their application to short RNAs and assessing one target molecule at a time.

The quantitative analysis of longer RNA fragments became possible by substituting gel electrophoresis with capillary electrophoresis [11]. However, major technology advancements occurred recently with the integration of probing methods and high-throughput sequencing technologies. This combination enabled the assessment of the structures of thousands of RNA molecules simultaneously, allowing for transcriptome-wide investigations of RNA structures [12], [13], [14], [15]. These breakthroughs gave rise to next-generation structure probing methods and spurred a wave of RNA “structurome” studies aimed at elucidating the functional impacts of RNA structure on the entire lifespan of all RNA molecules, including transcription [16], splicing [[12], [17]], processing [18], translation [[13], [17], [19]], degradation [[19], [20]], and beyond, all of which was previously experimentally challenging.

These high-throughput methods employ various enzymatical or chemical probes and readout strategies, providing us with a wealth of (albeit lower-resolution) structural information compared to biophysical methods like NMR and X-ray crystallography [21], [22], [23], [24], [25]. Based on the structural information obtained, these methods can be roughly categorized into two groups: *one-dimensional probing methods* that provide data about the local flexibility of nucleotides, indicating their propensity to form base pairs, and *two-dimensional probing methods* that reveal which RNA fragments are base-paired or interacting within close spatial distance.

**Fig. 1:**
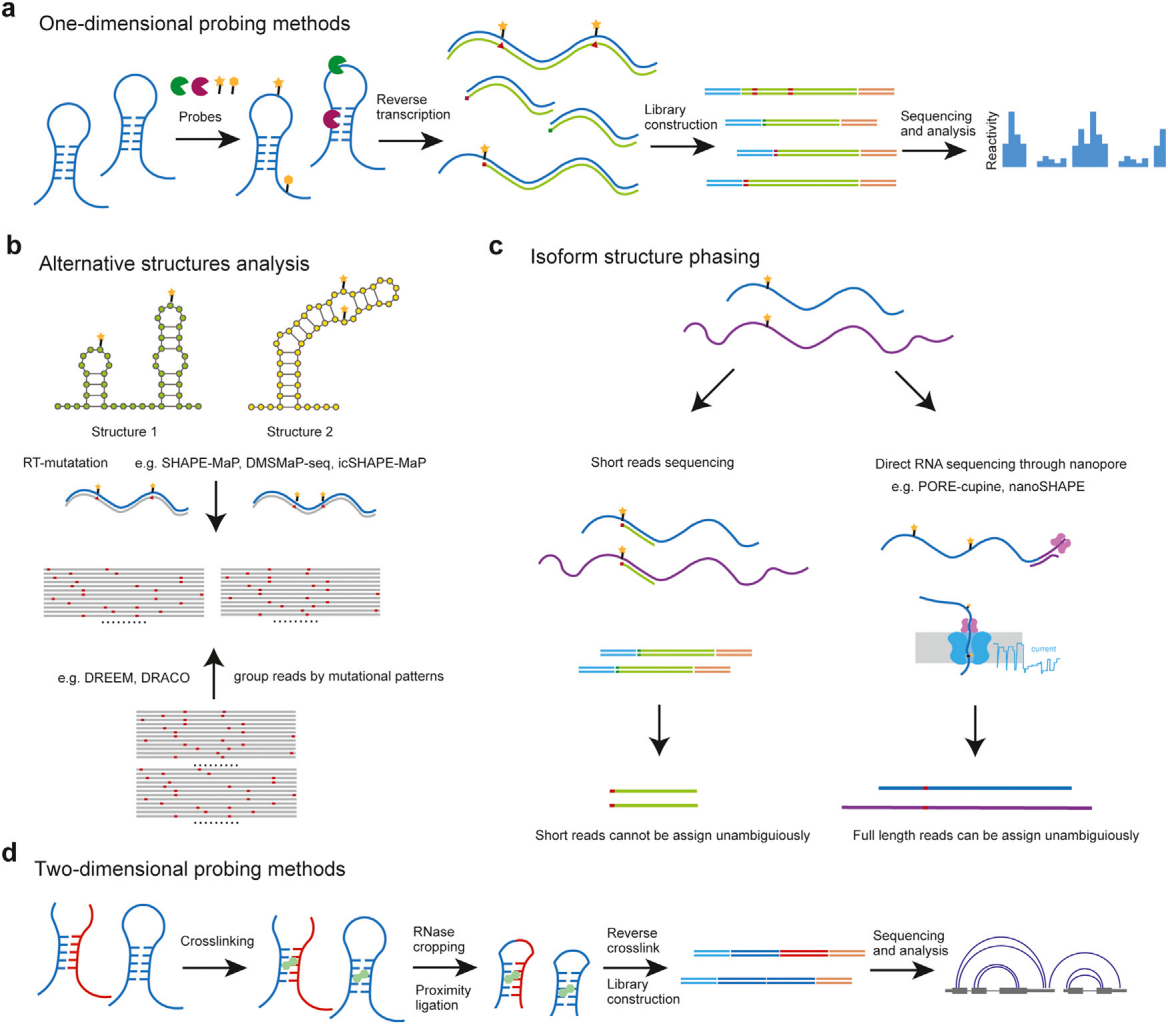
**RNA structure probing methods.** (A) General steps in one-dimensional methods to probe RNA structure. (B) Alternative structures of RNA can be inferred from chemical modification-induced mutations in reads. (C) Long read sequencing enables structural analysis of RNA isoforms. (D) Steps in two-dimensional structural probing methods.

*One-dimensional probing methods* employ enzymatic or chemical probes that cleave or modify nucleotides in a structure-specific manner. The cleavage or modification sites are then detected by reverse transcription, leading to the recording of truncations or mutations in cDNA, which can be analyzed through sequencing (Fig. 1a). Enzymatic probes like nuclease S1 and V1 selectively cleave single- and double-stranded regions, respectively, but are limited to *in vitro* structure probing due to their inability to penetrate the cell membrane [15]. Chemical probes have become a predominate approach, divided into base-specific probes and backbone-targeting probes. The base-specific probe dimethyl sulfate (DMS) [[12], [13], [26]] and the backbone-targeting SHAPE reagents (e.g., 1M7 [27] and NAI [[14], [28]]) are the most commonly used probes because they allow the assessment of *in vivo* structures. These probes have been successfully applied in studying the RNA structuromes in bacteria [29], plants [12], yeast [13], and mammalian cells and embryos [[19], [20]], and in the structural modeling of RNA viral genomes [30], [31], [32] and lncRNAs [[33], [34]].

More recent advances in this area have come from multiple research directions. New chemical probes are being developed to adapt to different application scenarios. For example, 1-ethyl-3-(3-dimethylaminopropyl)carbodiimide (EDC) was developed to profile G and U bases *in vivo* [[35], [36]], complementing DMS which primarily modifies single-stranded A and C bases. Another probe, 2A3 was suggested to be more reactive and accurate than NAI in capturing RNA structure *in vivo* [37].

In contrast to characterizing the ensemble average of RNA structures, new technologies have emerged to dissect their heterogeneous conformational states. Mutation-prone reverse transcription generates reads with multiple mutations, enabling the inference of different conformational states computationally [[38], [39]]. For example, DMS-MaPseq was developed to analyze alternative conformational states at splice sites in the RNA genomes of HIV-1 [38] and SARS-CoV-2 [40] (Fig. 1b).

It is important to note that most of these methods rely on short-read sequencing, which complicates the analysis of full-length RNA molecules and the characterization of isoform structures. Recent efforts have combined chemical probing with single molecule sequencings, such as the PORE-cupine [41] and nanoSHAPE [42] methods, enabling informative phasing of different RNA isoform structures (Fig. 1c). For example, the diverse conformational states of COOLAIR long non-coding RNA (lncRNA) isoforms were shown to change in response to external conditions [43].

Efforts have also been made to reduce the starting material requirement for RNA structure analysis. By focusing on improvements during library construction, such as on-bead reactions, the smartSHAPE method can profile the structural landscape of RNA using tens of thousands of macrophages [44]. It is anticipated that these technologies will continue to evolve, enabling RNA structure studies in rare specimens, such as early-development embryos or clinical samples. Furthermore, methodological advancements are likely to facilitate the profiling of extremely low-abundance transcripts, and even RNA structure analysis at the single-cell level.

*Two-dimensional probing methods* involve crosslinking and ligating RNA fragments that are in close spatial proximity (i.e., involved in base-pairing or other interactions). The ligated fragments are then subjected to library construction and sequencing, with the interacting fragments identified through data analyses (Fig. 1d). For example, several methods employ psoralen (or its derivatives) and focus on capturing base-pairing mediated interactions [45], [46], [47], [48]. These methods typically enrich crosslinked fragments using strategies like 2-D gel purification [45], biotin-psoralen [47], or antisense oligonucleotide pull-down [46] to increase specificity. However, they may still suffer from low efficiency of proximity ligation and crosslinking, laborious library construction protocols, and false signals reflecting spurious ligations. It is worth noting that psoralen has a bias for crosslinking staggered uridines [49].

Recently, the PARIS2 method improved upon the original PARIS method by utilizing the crosslinker amotosalen, optimizing protocols for 2D-gel RNA purification, and using singlet quenchers to prevent photochemical damage [50]. The SHARC method [25] uses DPI as a different crosslinker, which can react with 2′-OH groups of single-stranded nucleotides in proximity, dramatically increasing crosslinking efficiency to over 90%. SHARC also uses exonuclease trimming to enhance the precision of the two interacting fragments. SHAPE-JuMP [51] is a method that uses TBIA, a crosslinker similar to DPI, to crosslink two RNA fragments that are in close proximity. Additionally, it utilizes an engineered reverse transcriptase that can "jump" from one arm of the crosslinked fragments to the other, enabling the capture of the two interacting fragments. This innovative approach eliminates the requirement for a separate proximity ligation step.

Neither *one-dimensional* nor *two-dimensional probing methods* directly yield a complete structural model. However, they provide valuable information that can be used to constrain computational RNA structural modeling. For example, reactivity data obtained from *one-dimensional probing methods* can be converted into pseudo-energy terms and incorporated into energy or statistical models, as with RNAstructure [21]. This enables the modeling of secondary structures for various RNA molecules, including Xist [34] and the RNA genomes of ZIKA [31], HIV-1 [30], and SARS-CoV-2 [32]. Reactivity data can also help select the best-matched secondary structure models from a set of computationally generated models, as demonstrated by SeqFold [23]. Recent methods based on reverse transcription mutation have further enabled the characterization of alternative conformations of RNA molecules [38], [39], [40]. Interaction fragments identified by *two-dimensional probing methods* can be transformed into pairing probabilities to help algorithms select the most likely secondary structure models, as with IRIS [52].

In addition to facilitating secondary structure modeling, the obtained structural information can provide insights into higher-order RNA structures. For example, RING-MaP [53], MOHCA [54], and mutate-and-map [55] employ correlated chemical reactivities to infer higher-order RNA interactions and provide fuzzy distances. Furthermore, SHAPE-JuMP [51] directly identifies RNA interactions along with fuzzy distances, which can be used as constraints during 3D modeling. SHARC provides more accurate measurements of spatial distances between interacting fragments, enhancing Rosetta-based 3D modeling [25].

Despite these remarkable advancements, there remains a need for probing methods that offer more precise structural information with high spatial and temporal resolution. Similarly, powerful computational methods are required to effectively exploit such structural information, ultimately providing us with an unprecedentedly dynamic and high-resolution view of RNA structures *in vivo*.

## 3 X-ray crystallography

X-ray crystallography is a traditional and widely-used method for analyzing the structure of biomacromolecules. One of its key advantages is that it can determine the structure of molecules without size limitations and often yields high-resolution information. Over its more than 100 years of development, X-ray crystallography has become a well-established technique, benefitting from advancements such as automatic crystallization screening, synchrotron facilities with brilliant X-ray light, and a wealth of software tools for data collection, processing, and structure determination. In the field of RNA structure determination, X-ray crystallography remains the dominant technique, accounting for approximately 62% of the 1663 RNA-only structures deposited in the PDB. To perform XRC analysis, biomacromolecules need to be crystallized through screening precipitants, salts, temperatures, and sample concentrations. Various commercial crystallization kits, including those specifically designed for nucleic acids, can facilitate the initial screening process. Once crystals are obtained, they are subjected to X-ray diffraction, typically at synchrotron facilities, to measure the amplitudes of structural factors. To calculate electron densities by Fourier synthesis, the phases of structural factors that cannot be measured need to be determined through indirect approaches, known as the phasing problem.

Two critical steps that can pose challenges in X-ray crystallography are obtaining high-quality crystals and determining the phases for structure determination. Crystallization can be particularly challenging when working with RNA molecules, especially larger ones, as they tend to be inherently flexible and may not readily crystallize or form crystals with good diffraction properties [56].

In cases where homologous structures are available, it is possible to employ a technique called molecular replacement to determine the phases. Molecular replacement involves using a known structure as a searching template to solve the phase problem. The recent development of AlphaFold2, a deep learning-based method for protein structure prediction, has shown promising results in accurately predicting protein structures [57]. These predicted structures are often accurate enough to be utilized as search templates for molecular replacement. However, the accurate prediction of RNA 3D structure is still an ongoing challenge, and existing methods may not provide sufficient accuracy [[58], [59]].

In RNA crystallography, experimental phasing is often required due to the limited availability of homologous structures. De novo phasing relies on the use of heavy atom derivatives, which produce slightly different diffraction data compared to native crystals and generate anomalous signals. Various experimental methods, such as single-wavelength anomalous dispersion (SAD), multi-wavelength anomalous dispersion (MAD), and single isomorphous replacement with anomalous scattering (SIRAS), are employed to measure and utilize these signals to solve the phasing problem.

**Fig. 2:**
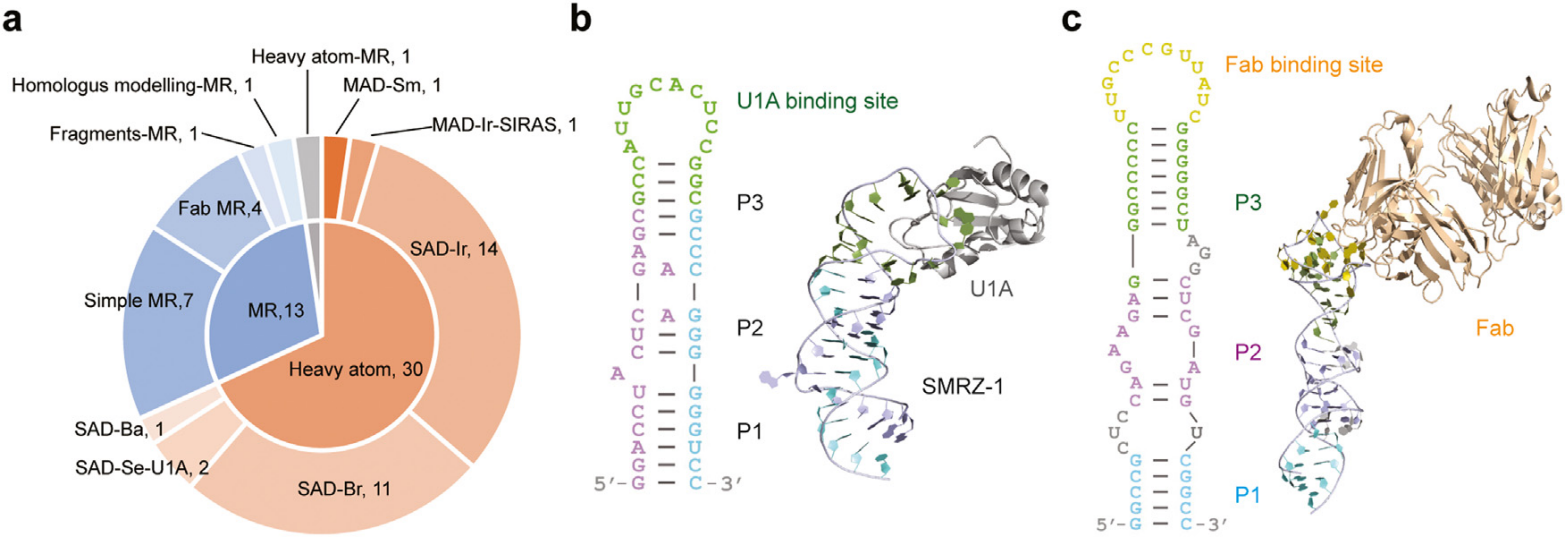
**X-ray crystallography analysis of RNA structure.** (A) Statistics of phasing methods for RNA crystal structures determined during 2017–2022. The inner circle shows the cases of molecular replacement (MR) and de novo phasing based on heavy atom derivatives. The outer circle further indicates the phasing method (SAD, MAD, SIRAS), the heavy atom used for de novo phasing, and the searching model for MR. (B) Crystal structure of SMRZ-1 RNA was determined in complex with U1A (PDB 7DLZ). (C) Crystal structure of SAR RNA was determined in complex with Fab HAVx (PDB 6XJY).

To introduce heavy atoms into RNA crystals, soaking methods are commonly used. Iridium has emerged as a popular choice and has been utilized to phase nearly half of the recently determined RNA structures (Fig. 2a, Table S1). The soaking process can be facilitated by engineering GU wobble pairs in RNA helices, as they provide high-affinity binding sites for trivalent hexamine complex ions, such as cobalt, iridium, and osmium [[60], [61]]. Alternatively, heavy atoms can be covalently incorporated during the chemical synthesis of RNA. This method is similar to incorporating Se-labeled methionine (Se-Met) into recombinantly expressed proteins. However, this approach is limited to RNA molecules smaller than ∼60 nt in length, as chemical synthesis becomes challenging beyond this size range. Bromo-substituted nucleosides are frequently used in RNA crystallography (Fig. 2a, Table S1).

Incorporating RNA-binding proteins is another strategy to facilitate the packing and phasing of RNA crystals. Phases can be obtained when the introduced protein is labeled with Se-Met or the protein can serve as a search template for molecular replacement. One protein commonly used in this approach is U1A, which contains an RNA-recognition motif (RRM) and tightly binds specific sequences in a hairpin loop. By engineering an U1A-binding sequence that can be recognized and bound by U1A, dozens of RNA structures have been successfully solved [62] (Fig. 2b, Table S1). Fab fragments of antibodies that bind specific RNAs have also been employed to assist in the crystallization and phasing of RNA structures [[63], [64]]. Fab has a larger molecular weight (50 kDa) compared to U1A (11 kDa), providing an additional option to facilitate the formation of crystal packing interaction and phasing by molecular replacement. Furthermore, large Fab-RNA complexes are also suitable for cryo-EM analysis. Several RNA structures have been recently determined using Fab co-crystallization and molecular replacement methods [65], [66], [67], [68], [69] (Fig. 2c, Table S1).

## 4 Nuclear magnetic resonance spectroscopy

NMR is a well-established technique widely utilized for the determination of the structure, dynamics, and interactions of biomolecules, including RNAs [[70], [71]]. Since the first NMR structure of a stable 12-nt RNA hairpin was determined in 1990 [72], a large number of RNA structures have been elucidated using NMR. As of October 2022, approximately 587 NMR structures of RNA have been deposited in the PDB, constituting around 35% of the total 1663 RNA-only structures available. NMR spectroscopy also enables the investigation of structural changes in RNA induced by ligand binding, alternations in buffer or environmental conditions, and the characterization of RNA dynamics across a broad range of time scales, spanning from picoseconds to hours [73].

NMR spectroscopy has traditionally encountered challenges when studying large RNA molecules, primarily due to the deteriorated spectral resolution and reduced sensitivity that arise with increasing RNA length [[74], [75]]. As a result, NMR is more suitable for investigating small to medium-sized RNAs, typically ranging from approximately 12 to 50 nt. Out of the 587 NMR structures of RNA deposited in the PDB, the average size is around 30 nt, with only 26 and 8 RNAs exceeding 50 and 100 nt in length, respectively (Table S1). However, significant progress has been made in biomolecular NMR spectroscopy in recent years, enabling the investigation of larger RNA molecules up to 200–300 nt in length [[74], [76], [77]]. These advancements have been facilitated by several key developments in NMR instrumentation, RNA sample preparation, data acquisition, and data analysis. These include the commercial availability of high-field magnets with up to 1.2 GHz ^1^H Larmor frequency (28.2 T) [78], the introduction of cryogenically cooled probes [79], the implementation of non-uniform sampling and associated processing algorithms [80], novel strategies for RNA synthesis and selective isotope labeling [[74], [75], [77], [81]], the design of tailored NMR experiments for specific isotope labeling schemes [[82], [83]], and the automation of resonance assignment and structure calculation [[84], [85]]. The ongoing development of NMR methods has been accompanied by many significant applications of NMR in determination of both 2D and 3D structures of large RNAs.

**Fig. 3:**
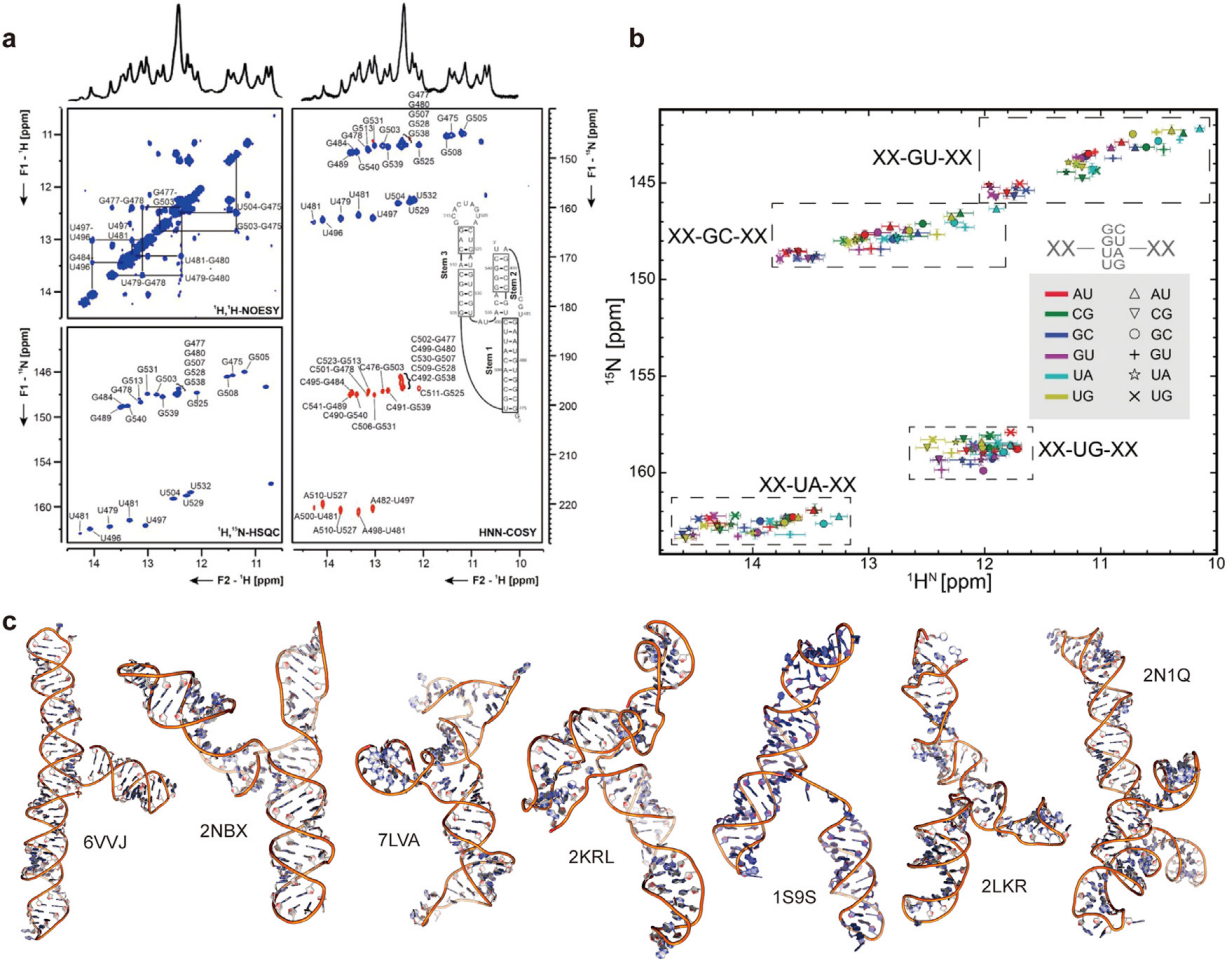
**NMR spectroscopy as a tool for 2D and 3D structure determination of large RNAs.** (A) The 2D structure of the ribosomal frame shifting signal RNA, a conserved three-stemmed pseudoknot (PK) structure in the genomic RNA of SARS-CoV-2 was inferred from imino-proton correlation by a combination of ^1^H,^1^H-NOESY, ^1^H,^15^N-TROSY and HNN—COSY spectra. Reprinted, with permission, from Reference [89]. (B) The average imino resonance of each base-pair (BP) triplet is shown as a marker on the imino chemical shift map of BP triplets [88]. The dashed boxes depict the chemical shift distribution range of the central guanine or uridine residue. (C) 3D structures of RNAs exceeding 100 nt were determined using NMR spectroscopy, more detailed description about these RNAs is found in Table S1.

NMR offers a powerful tool for determining RNA secondary structure. Unlike chemical probing experiments that provide one-dimensional reactivities per nucleotide without explicit information about base pairing, NMR experiments can detect hydrogen bonding between base pairs. This enables the identification of the number and type of base pairs, as well as their sequential neighbors [[86], [87]].The imino protons of G and U involved in base pairing exhibit distinct NOE patterns and large chemical shifts in the range of 10 to 15 ppm, making them characteristic “fingerprints” of RNA secondary structure (Fig. 3a). Accurate chemical shift assignments are essential for quantitative NMR analysis such as RNA secondary structure determination. In the case of small RNAs, resonance assignment strategies primarily rely on NOEs. The imino-imino sequential walk within the imino region of 2D NOESY spectra allows for the identification of connectives between AU, GC, and GU base pairs in helical regions [70]. The ^1^H^N^ and ^15^N imino chemical shifts for RNA helical segments composed of only WC base pairs and GU wobbles can be accurately predicted using a base pair triplet lookup table (Fig. 3b) [88]. These prediction tools can facilitate or validate imino resonance assignments and aid in RNA secondary structure determination.

The NMR-based procedure for RNA 3D structure determination generally includes isotope labeling of RNA samples, buffer and temperature optimization, acquisition of NMR spectra, resonance assignment, collection of structural constraints, and structure calculation and validation [[70], [74], [90]]. While most NMR-solved RNA structures are small, recent advancements in NMR methods have allowed for the determination of larger RNA structures exceeding 100 nt (Fig. 3c) [[91], [92], [93], [94], [95], [96], [97], [98]], with the 155-nt HIV-1 packaging signal being the largest reported [97].

Resonance assignments in RNA, especially for large molecules, are challenging due to limited chemical shift dispersion. To address this, nucleotide-specific isotope labeling techniques are employed using atom-specific ^13^C/^15^N/^2^H/^19^F labeled nucleotides in in vitro transcription [[75], [83]]. Segmental isotope labeling strategies have been developed to simplify the spectra of large RNAs. Currently, analysis of NOESY spectra is the primary method for RNA spectral assignments, focusing on proton detection. However, proton-detected experiments face difficulties in studying large RNAs due to low proton densities of nucleobases and poor chemical shift dispersion. To extend the NMR size limitation and to characterize large RNAs, low-γ heteronuclei-detected (like ^13^C or ^15^N) experiments involving through-bond correlations of nucleobases and the phosphodiester backbone of RNA for chemical shift assignment have been developed [82].

After achieving unambiguous resonance assignments, the determination of RNA structure relies on sufficient structural constraints [[74], [90]]. Hydrogen bonding constraints are particularly powerful in defining RNA secondary structure, as they constrain the bases to linear and planar arrangements. Large RNAs often exhibit elongated structures, resembling cylindrical shapes rather than globular ones. Due to the lack of NOE information between distant ends of the molecule, the relative orientations of helical segments at opposite ends can be poorly defined. Integration of global structural constraints, such as residual dipolar couplings (RDC), which provide unique angular restraints, significantly enhances the precision of NMR structures at both local and global levels [[77], [99]]. Long-range distance constraints derived from paramagnetic NMR effects can also be incorporated into RNA structure calculation. Recently, Tjandra and Summers developed a general approach to induce paramagnetic effects on RNA via a paramagnetically tagged protein, allowing for obtaining long-range RNA structural constraints through Lanthanide-induced pseudocontact shifts [100]. Ongoing advancements in NMR methods for acquiring RDCs and other global restraints hold promise for further expanding the size limit of RNA 3D structure determination.

## 5 Cryo-electron microscopy

Cryo-EM offers the advantage of directly visualizing macromolecules without the need for crystallization and phasing, as required in X-ray crystallography. In cryo-EM single-particle reconstruction, 2D projections of particles are recorded from numerous orientations, and these projections are then used to reconstruct 3D structures. Thanks to recent advancements in hardware and software, cryo-EM has enabled the determination of atomic-resolution structures of biomacromolecules, making it the most powerful tool available for structural biologists. However, obtaining high-resolution (< 3 Å) cryo-EM maps for RNA molecules remains challenging due to their small size and flexibility.

Hybrid approaches have been developed to overcome the limitations of cryo-EM for high-resolution RNA structure determination. These approaches involve combining cryo-EM maps at intermediate resolution with structures obtained with other techniques such as NMR, crystallography, and theoretical modeling (Table S1). For example, the structure of the HIV-1 Dimer Initiation Site (DIS) dimer, consisting of 94 nt, was determined by combining a cryo-EM map at ∼9 Å resolution and NMR distance restraints [101]. This hybrid model confirmed that the bulges in the cryo-EM density were caused by guanine bases being flipped out. T-box is a regulatory RNA element present in bacterial mRNAs that binds to non-aminoacylated tRNAs upon amino acid starvation. The full-length complex of the *B. subtilis* glyQS T-box and tRNA^Gly^ was analyzed by cryo-EM, resulting in an overall resolution of 4.9 Å. However, the peripheral region of the T-box could only be resolved to 6 Å, which hindered *de novo* model building. To overcome this limitation, a combination of crystal structures of subcomplexes and the cryo-EM map was used to model the entire T-box-tRNA complex [102]. This hybrid model provided insights into the structural basis of amino acid sensing through higher-order RNA-RNA interactions. Another recent study showcased the power of combining cryo-EM and X-crystallography for RNA structure determination [103]. In this study, cryo-EM structures of the double element for nuclear expression (dENE) were resolved before and after binding poly(A) tail, at resolutions of 8.7 Å and 5.6 Å, respectively. A computational atomic model derived from the high-resolution crystal structure of the dENE+poly(A)_28_ complex was then fitted to the cryo-EM map using real-space refinement [104]. The cryo-EM structure not only confirmed the crystal structure but also provided additional insight into the interactions between dENE and poly(A). These examples highlight the effectiveness of hybrid approaches, combining cryo-EM with other techniques, to enhance the determination of RNA structures and provide valuable insights into their functional mechanisms.

**Fig. 4:**
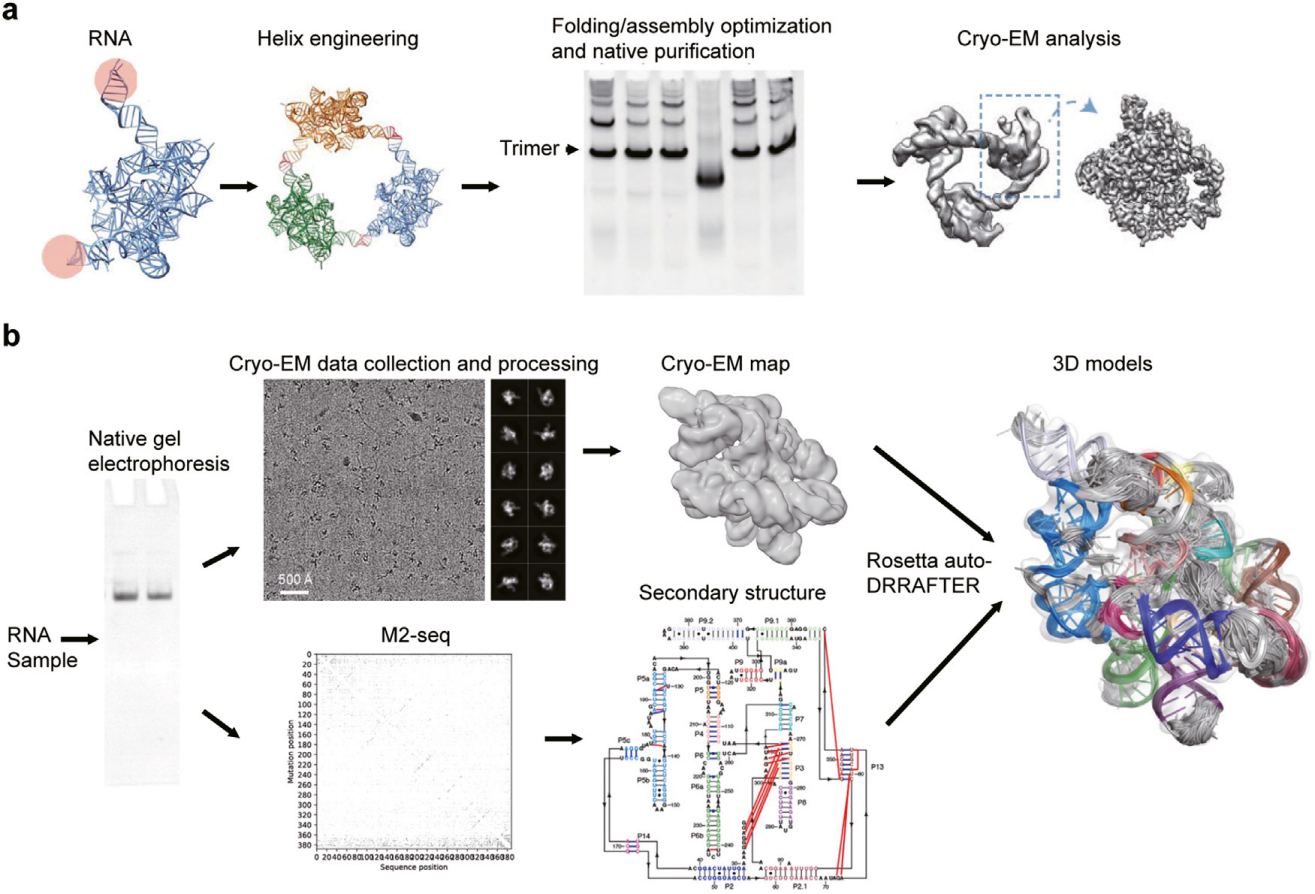
**Cryo-EM analysis of RNA structures.** (A) Workflow of ROCK [105]. (B) Pipeline of Ribosolve [106].

Symmetric oligomers have certain advantages when it comes to cryo-EM analysis. Due to their larger sizes and the ability to average symmetry-related volumes, they often yield better density. To facilitate cryo-EM analysis of RNA, a nano-architectural engineering strategy called ROCK (RNA oligomerization-enabled cryo-EM via installing kissing loops) has been developed [105] (Fig. 4a). This technique involves the insertion of kissing loop sequences into functionally non-essential regions of the RNA molecules, enabling the self-assembly of the RNA into symmetric ring-shaped oligomers. ROCK has been successfully applied in determining the structures of several RNAs at near-atomic to subnanometer resolutions, including *Tetrahymena* and *Azoarcus* group I introns and an FMN riboswitch.

The Ribosolve pipeline was recently developed to efficiently resolve cryo-EM structures of RNA at medium resolution [106] (Fig. 4b). The pipeline encompasses several steps, including native gel electrophoresis of RNA, determination of RNA secondary structure using M2-seq, cryo-EM single-particle reconstruction and automatic RNA modeling using Rosetta auto-DRRAFTER. Native gel electrophoresis is used to rapidly determine the optimal folding conditions of the RNA of interest. RNA that forms a single band in native gels is subjected to M2-seq to assess the conformational homogeneity and to assist RNA modeling in the late stages. The RNA sample is then subjected to cryo-EM single-particle reconstruction, which often yields medium resolutions ranging from 5 to 11 Å. At this resolution range, the major and minor grooves of RNA helices become discernible, but *de novo* modeling remains challenging. To overcome the difficulties in modeling, the Rosetta auto-DRRAFTER algorithm is employed. The algorithm leverages the measured secondary structures and cryo-EM density maps to automatically generate atomic models of the RNA.

Ribosolve has been applied to determine over ten RNA structures (Table S1). The structures of SAM-IV riboswitches were determined in both the apo and ligand-bound states at resolutions of 3.7 Å and 4.1 Å, respectively [107]. These structures revealed similarities in the ligand binding region to other homologous SAM-I and SAM-I/IV riboswitches, but also showed a more complex peripheral structure that likely plays a role in regulating SAM binding. Ribosolve was also applied to solve the structure of the 28-kDa SARS-CoV-2 frameshift element (FSE) at 6.9 Å resolution [108]. This structure guided the development of antisense oligonucleotides to inhibit FSE-mediated frameshift function and viral replication. For the full-length *Tetrahymena* ribozyme, Ribosolve initially solved its apo state structure at 6.8 Å resolution [106]. Subsequent cryo-EM analysis, involving a larger number of particles, enables the determination of the apo, substrate-bound and misfolded states at resolutions of 3–4 Å [[109], [110]]. These high-resolution structures of the *Tetrahymena* ribozyme revealed important long-range tertiary interactions between the peripheral and core regions of the ribozyme, significant conformational rearrangements upon the binding of the internal guide sequence to the substrate, and the identification of 31 critical metal ions for proper RNA folding and catalytic activity. These studies provide structural explanations for the extensive biochemical study conducted over four decades on the splicing reaction of the *Tetrahymena* ribozyme.

Cryo-EM structure determination of RNA sometimes benefits from the introduction of structure-stabilizing RNA-binding proteins. This approach was exemplified by the cryo-EM structure of L. *lactis* L1.LtrB group II intron, which improved from 4.5 Å to 3.8 Å upon binding to its intron-encoded protein partner, LtrA [111]. However, this approach should be used with caution, as protein binding can potentially induce unexpected conformational changes in RNA molecules. In summary, cryo-EM-centric approaches can accelerate the structure determination of RNA molecules with critical biological and biomedical significance.

## 6 Small-angle X-ray scattering

In the last two decades, the field of SAXS has experienced significant advancements, including the availability of high-brilliance SAXS beamlines at synchrotron facilities, improved data analysis tools, and advancements in sample presentation and preparation techniques. These developments have established SAXS as a prominent technique for comprehensive analyses of biomolecular structure, dynamics, and interactions, including RNA molecules in solution [[112], [113]]. The basic procedure of a SAXS experiment involves illuminating a sample with an X-ray beam and recording the intensity and angle of the scattered X-rays. The resulting one-dimensional scattering curve contains valuable information about the particle in solution, including molecular mass, size, radius of gyration, aggregation state, folding state, flexibility, domain organization, and overall 3D shape [114].

SAXS was first utilized for RNA structure analysis in 2000 [115], where the shape of *Thermus flavus* 5S RNA was determined at a resolution of 1.3 nm using an *ab initio* simulated annealing algorithm [115]. Since then, SAXS has gained popularity as a valuable tool for the structural and biophysical characterization of RNA [113]. RNA, with its electron-rich sugar-phosphate backbone, exhibits strong X-ray scattering and can be studied at low concentrations using SAXS [116]. SAXS offers several advantages for RNA studies, including the ability to analyze molecules without the need for crystallization, suitability for a wide range of molecule sizes, and generation of low-resolution 3D topological structures under near-physiological conditions that are not easily accessible by other high-resolution structural techniques.

The applicability of SAXS for determining the topological structure of RNA is derived from several unique properties of RNA: (a) RNA secondary structure can be predicted and/or experimentally determined using chemical probing or NMR before SAXS analysis [117], (b) A-form duplexes, which are prevalent in RNA structures, can be confidently modeled at the atomic level [118], (c) A-form duplexes, with their characteristic cross-sectional diameter of ∼22 Å, can be readily identified within the *ab initio* reconstructed shape envelopes derived from SAXS data [119], (d) Large RNAs often possess a modular organization, making them amenable to divide-and-conquer approaches for structural studies [120], (e) The overall 3D shape of large RNAs tends to be planar rather than globular. In essence, RNA tertiary structures can be considered as an assembly of packed duplexes connected by single-stranded linkers, interrupted by internal loops or bulges, and capped with terminal loops. Once the orientations and positions of these duplexes are determined, the approximate topological structure of the RNA can be inferred [[121], [122]].

**Fig. 5:**
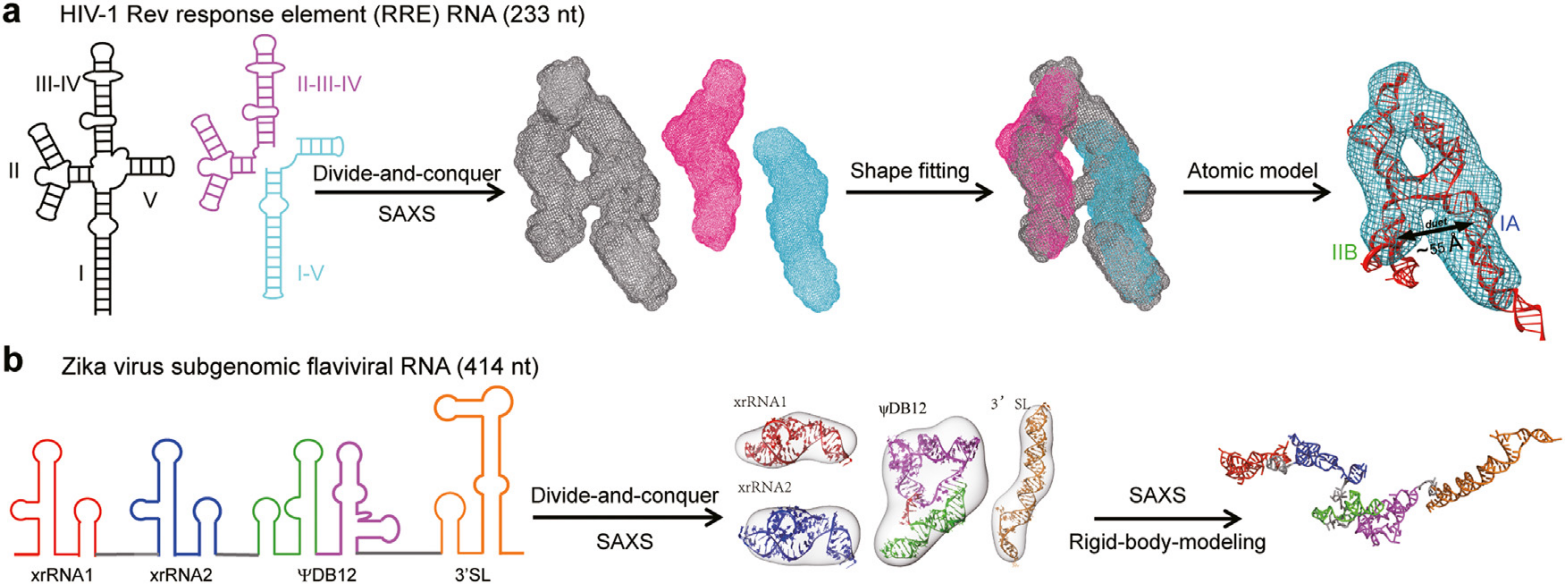
**SAXS as a tool for topological 3D structure determination of large RNAs.** (A) Illustration of general procedures for 3D structure determination of the HIV-1 Rev response element (RRE) RNA by using SAXS and computation [119]. Following the divide-and-conquer strategy, SAXS-derived *ab initio* 3D shape envelops were used to define the topology of the individual domains within the full length RNA. Such information was further used to guide the atomic modeling of the RNA. (B) Illustration of the general procedures for 3D structure determination of the Zika virus subgenomic flaviviral RNA (sfRNA) by using SAXS and computation [120]. Alternatively, SAXS data can be used as a filter to define the 3D structure of the individual subdomains within a modular large RNAs from the conformational pools generated using the secondary structure information and structure prediction programs. Atomic structure of the full-length RNA can be modeled by rigid-body modeling using SAXS data as restraints. Additional restraints can be incorporated into this process as well.

SAXS has been widely utilized for determining low-resolution 3D topological structures of large RNAs, employing two main approaches. The first approach involves using SAXS-derived molecular envelopes to identify the orientations and positions of duplexes within an RNA domain (Fig. 5a). For large modular RNAs with multiple domains, individual domains or structural components can be separately placed within the molecular envelope. Guided by the topological structural information, atomic models can then be built up and refined against the SAXS data. This approach has been utilized to derive the topological structures of various RNAs, such as the Varkud satellite (VS) ribozyme [123], the HIV-1 Rev response element RNA [119] and the T-box riboswitch core [124]. The second approach utilizes RNA secondary structure information to generate a pool of conformers using RNA 3D structure prediction programs. The SAXS data is then used as a filter to select conformers that match the experimental data. This approach has been applied to analyze the structures of diverse RNAs, including long non-coding flaviviral subgenomic RNAs (Fig. 5b) [120], the hepatitis C virus internal ribosome entry site RNA [125], the 3′X and 5BSL3.2 domains of HCV [126], the HIV-1 5′ UTR RNAs [127] and both the sense and antisense of human LincRNA-p21 [126]. Low-resolution 3D topological structures obtained through SAXS frequently provide valuable insights into RNA function [[119], [127]]. With the increasing availability of secondary structure information for large RNAs [128] and improvements in RNA structure prediction algorithms [118], the combination of SAXS and computational modeling can be a powerful technique for bridging the secondary and tertiary structure of large RNAs and promoting their structure*-*function relationship analysis.

## 7 Perspective

RNA molecules are often modular and adopt flexible or multiple structures, which imposes significant challenges for structural characterization by individual techniques. The data obtained from multiple approaches, physical theories and statistical analyses could be integrated to form a more comprehensive understanding of dynamic RNA structures. The concept of hybrid or integrative modeling, originally developed for protein complexes [129], can also be extended to RNA systems [130]. In addition to the techniques discussed earlier, there are other methods available to provide structural constraints for RNA. Electron paramagnetic resonance (EPR) spectroscopy, X-ray scattering interferometry (XSI), and single-molecule Förster resonance energy transfer (smFRET) can measure inter- or intra-molecular distances in the nanometer range for RNA molecules [[131], [132], [133]]. These techniques require site-specific labeling of RNAs with spin labels, gold nanoparticles, or fluorescent dyes [[134], [135], [136]]. Combining data from SAXS [[91], [92], [93], [121]], cryo-EM map [[101], [137]] and EPR distance restraints [138] with NMR can help refine NMR-derived RNA structural ensembles. The integrative structural biology approach, which combines information from diverse experimental and computational techniques, will continue to be needed in tackling difficult RNA structures.

Decades of structural study have demonstrated the ability of RNA to form intricate higher-order structures （Table S1) [[139], [140], [141], [142], [143], [144], [145], [146], [147], [148], [149], [150], [151], [152], [153], [154], [155], [156], [157], [158], [159], [160], [161], [162], [163], [164], [165], [166], [167], [168], [169], [170], [171], [172], [173], [174]]. However, compared to proteins, the number of known RNA sequences that adopt complex structures is relatively limited. The secondary structure of RNA can be now efficiently probed at the transcriptome level. There is a pressing need to develop high-throughput techniques that can efficiently identify and characterize higher-order structure elements in RNA sequences. Such developments may allow to discover (hopefully many more) new structural elements in mRNA and ncRNA and expand our understanding of the functionality of RNA structure.

## Declaration of competing interest

The authors declare that they have no conflicts of interest in this work.

## Appendix Supplementary materials

Image, application 1
