# Supplementary material for: RNA structure determination: From 2D to 3D
Source: Fundam Res. 2023 Jun 12;3(5):727–37. doi: 10.1016/j.fmre.2023.06.001 (PMC11197651; doi:10.1016/j.fmre.2023.06.001)
Supplement: Supplementary file 1 [file mmc1.pdf]

RNA structure determination: from 2D to 3D

Supplementary Materials: Table S1

Table S1. Recently determined important RNA structures.

RNA	Species	Length (nt)	Structure determination method[a]	Resolution (Å)	PDB or EMDB	Year	Reference
ykkC Guanidine-I riboswitch	*S. acidophilus*	95	XRC (MAD, Ir/SIRAS)	2.71	5T83	2017	[139]
ykkC Guanidine-I riboswitch	*D. dadantii*	85	XRC (SAD, Ir)	2.30	5U3G	2017	[140]
ykkC Guanidine-I riboswitch	*Burkholderia sp.*	128	XRC (MR, 5T83)	2.70	7MLW	2021	[141]
ykkC 2a riboswitch (ppGpp)	*S. acidophilus*	102	XRC (MR, 6DLR)	2.20	6DMC	2018	[142]
ykkC 2b riboswitch (PRPP)	*S. lipocalidus*	107	XRC (SAD, Ir)	2.66	6DLR	2018	[142]
ykkC 2b riboswitch (PRPP)	*T. mathranii*	117	XRC (MR, 5T83)	2.49	6CK5	2018	[143]
mini-*ykkC* Guanidine-II riboswitch P1-7bp	*G. violaceus*	18	XRC (SAD, Br)	1.69	5NEO	2017	[144]
mini-*ykkC* Guanidine-II riboswitch P2-6bp	*G. violaceus*	16	XRC (SAD, Br)	1.81	5NDH	2017	[144]
mini-*ykkC* Guanidine-II riboswitch P1-8bp	*E. coli*	20	XRC (SAD, Br)	2.57	5NDI	2017	[144]
mini-*ykkC* Guanidine-II riboswitch P2-6bp	*P. aeruginosa*	16	XRC (SAD, Br)	2.10	5VJB	2017	[145]
ykkC-III Guanidine-III riboswitch	*T. fusca*	41	XRC (SAD, Br)	1.91	5NWQ	2017	[146]
Glutamine-II riboswitch	*Prochlorococcus sp.*	50	XRC (MR, homologous model)	2.30	6QN3	2019	[147]
SAM-V riboswitch	*C. pelagibacter*	53	XRC (SAD, Br)	2.50	6FZ0	2018	[148]
SAM-VI riboswitch	*B. angulatum*	55	XRC (SAD, Se-U1A)	2.71	6LAS	2019	[149]
SAM-SAH riboswitch	*Roseobacter sp.*	35	XRC (SAD, Br)	1.70	6YL5	2020	[150]
NAD^+-I riboswitch (domain I)	*C. koribacter*	52	XRC (SAD, Br)	2.10	6TF0	2020	[151]
NAD^+-I riboswitch (domain I)	Metagenome	57	XRC (SAD, Se-U1A)	2.80	7D7V	2020	[152]
NAD^+-I riboswitch (domain II)	Metagenome	50	XRC (MR, 7D7V)	2.10	7D81	2020	[152]
Xanthine riboswitch	*Ideonella sp.*	46	XRC (SAD, Ir)	2.79	7ELP	2021	[153]
T-box riboswitch	*G. kaustophilus*	66+75	XRC (SAD, Ir)	2.66	6PMO	2019	[101]
T-box riboswitch	*M. tuberculosis*	166+77	XRC (SAD, Ir)	2.93	6UFG	2019	[154]
iSpinach fluorogenic RNA	Synthetic	69	XRC (MR, 4Q9R)	2.00	5OB3	2017	[155]
Corn fluorogenic RNA	Synthetic	36	XRC (SAD, Ir)	2.51	5BJP	2017	[156]
Mango fluorogenic RNA	Synthetic	31	XRC (SAD, Ir)	1.70	5V3F	2017	[157]
DIR2s fluorogenic RNA	Synthetic	60	XRC (MR, Fab)	1.87	6DB8	2018	[67]
Mango-II fluorogenic RNA	Synthetic	36	XRC (MR, 5V3F)	2.90	6C63	2018	[158]

Mango-III fluorogenic RNA	Synthetic	37	XRC (SAD, Ir)	1.55	6E8U	2019	[159]
iMango-III fluorogenic RNA	Synthetic	38	XRC (MR, 6E8U)	2.35	6E8S	2019	[159]
Mango-IV fluorogenic RNA	Synthetic	28	XRC (SAD, Br)	2.40	6V9B	2020	[160]
Chili fluorogenic RNA	Synthetic	52	XRC (SAD, Ir)	2.80	7OA3	2021	[161]
Pepper fluorogenic RNA	Synthetic	48	XRC (SAD, Ir)	1.50	7EOG	2021	[162]
Pepper fluorogenic RNA	Synthetic	67	XRC (MR, Fab)	2.24	7SZU	2022	[69]
Squash fluorogenic RNA	Synthetic	83	XRC (SAD, Ir)	2.73	7KVT	2022	[163]
Twister-sister ribozyme	Metagenome	40+22	XRC (SAD, Br)	2.00	5T5A	2017	[164]
Twister-sister ribozyme	Metagenome	50+18	XRC (SAD, Ir)	2.00	5Y85	2017	[165]
Pistol ribozyme	Metagenome	50+15	XRC (MAD, Sm)	2.97	5KTJ	2017	[166]
Pistol ribozyme	*A. putredinis*	50+15	XRC (SAD, Br)	3.10	6R47	2019	[167]
Hatchet ribozyme	Metagenome	81	XRC (SAD, Ir)	2.63	6JQ6	2019	[168]
SAM ribozyme-1	Synthetic	45	XRC (MR, U1A)	3.00	7DLZ	2021	[169]
Self-alkylating ribozyme (SAR)	Synthetic	58	XRC (MR, Fab)	2.16	6XJY	2021	[68]
Methyltransferase-ribozyme 1 (MTR1)	Synthetic	14+ 24+24	XRC (MR, fragments)	2.80	7Q7X	2022	[170]
MTR1	Synthetic	68	XRC (SAD, Ba)	2.30	7V9E	2022	[171]
Frameshifting pseudoknot	*SARS-CoV-2*	65	XRC (SAD, IR/ MR, Fab)	2.09	7MLX	2021	[172]
Frameshifting pseudoknot	*SARS-CoV-2*	66	XRC (SAD, Ir)	1.31	7MKY	2022	[173]
HIV-1 core packaging signal	HIV-1	155	NMR (NOE, angles, H-bonds)		2N1Q	2015	[96]
A conserved retroviral RNA packaging element	Moloney murine leukemia virus	131	NMR (NOE, angles, H-bonds, cryo-EM)		2L1F	2010	[97]
Cap1G-TPUA	HIV-1	130	NMR (NOE, angles, H-bonds)		6VVJ	2020	[93]
Yeast U2/U6 complex	*S. cerevisiae*	111	NMR (NOE, angles, RDC, H-bonds, SAXS)		2LKR	2011	[91]
J-K region of EMCV IRES	Synthetic construct	108	NMR (NOE, angles, H-bonds, SAXS)		2NBX	2016	[92]
HIV-1 PBS-segment	HIV-1	103	NMR (NOE, angles, H-bonds, SAXS)		7LVA	2021	[94]
Ribosome binding structure element	Turnip crinkle virus	102	NMR (H-bond, RDC, SAXS)		2KRL	2009	[90]
MLV PSI site	Moloney Murine Leukemia Virus	101	NMR (NOE, angles, RDC, H-bonds)		1S9S	2004	[95]
Dimer initiation site (DIS)	HIV-1	94	CryoEM-NMR	~9	6BG9	2018	[100]
glyQS T-box-$tRNA^{Gly}$	*B. subtilis*	244	CryoEM-crystallography	4.9	6POM	2019	[101]
dENE	Rice TWIFB1	76	CryoEM-crystallography	8.7	EMD-23 402	2021	[102]

dENE-poly(A)$_{28}$	Rice TWIFB1	104	CryoEM-crystallography	5.6	7LJY	2021	[102]
Apo L-21 ScaI ribozyme	*Tetrahymena*	388	CryoEM (Ribosolve)	6.8	6WLS	2020	[105]
hc16 ligase product		349	CryoEM (Ribosolve)	10	6WLN	2020	[105]
hc16 ligase		338	CryoEM (Ribosolve)	11	6WLO	2020	[105]
Glycine riboswitch with glycine	*V. cholerae*	231	CryoEM (Ribosolve)	5.7	6WLU	2020	[105]
Apo Glycine riboswitch	*V. cholerae*	231	CryoEM (Ribosolve)	4.8	6WLT	2020	[105]
Glycine riboswitch with glycine	*F. nucleatum*	171	CryoEM (Ribosolve)	7.4	6WLM	2020	[105]
Apo glycine riboswitch	*F. nucleatum*	171	CryoEM (Ribosolve)	10	6WLL	2020	[105]
ATP-TTR-3 with AMP		130	CryoEM (Ribosolve)	9.6	6WLJ	2020	[105]
Apo ATP-TTR-3		130	CryoEM (Ribosolve)	10	6WLK	2020	[105]
SAM-IV riboswitch with SAM	*Mycobacterium*	119	CryoEM (Ribosolve)	4.8	6WLR	2020	[105]
Apo SAM-IV riboswitch	*Mycobacterium*	119	CryoEM (Ribosolve)	4.7	6WLQ	2020	[105]
SAM-IV riboswitch with SAM	*Mycobacterium*	119	CryoEM (Ribosolve)	4.1	6UET	2019	[106]
Apo SAM-IV riboswitch	*Mycobacterium*	119	CryoEM (Ribosolve)	3.7	6UES	2019	[106]
L-16 ScaI ribozyme with substrates	*Tetrahymena*	407	CryoEM (Ribosolve)	3.1	7EZ2	2021	[109]
Apo L-21 ScaI ribozyme	*Tetrahymena*	388	CryoEM (Ribosolve)	3.1	7EZ0	2021	[109]
Frameshifting pseudoknot (FSE)	SARS-CoV-2	88	CryoEM (Ribosolve)	6.9	6XRZ	2021	[107]
Group I intron	*Tetrahymena*	388	CryoEM (ROCK)	3.0-4.2	7R6L, 7R6M, 7R6N	2022	[104]
Group I intron	*Azoarcus*	197	CryoEM (ROCK)	4.9	EMD-24284	2022	[104]
FMN riboswitch with FMN	*F. nucleatum*	112	CryoEM (ROCK)	5.9	EMD-24285	2022	[104]
L1.LtrB group II intron	*L. lactis*	702	CryoEM	4.5	5G2Y	2016	[110]
tRNA-like structure	Brome mosaic virus	169	CryoEM	4.3	7SAM	2021	[174]

a. For structures determined by X-ray crystallography (XRC) during 2017-2022, the phasing method and the used heavy atoms are listed: Single-wavelength anomalous dispersion (SAD), Multi-wavelength anomalous dispersion (MAD), Single isomorphous replacement with anomalous scattering (SIRAS), Molecular replacement (MR) and searching model. For structures determined by NMR, the types of restraint used in structure calculation are listed. For structures determined by cryo-EM, the additional hybrid methods are listed.
